# Intimate Partner Violence, prevalence and its consequences: a community-based study in Gambella, Ethiopia

**DOI:** 10.3389/fpubh.2024.1412788

**Published:** 2024-05-27

**Authors:** Abdi Geda Gedefa, Tsegaye Abdi, Desalegn Chilo, Gebiso Roba Debele, Ayantu Girma, Misra Abdulahi

**Affiliations:** ^1^Public Health Departments, College of Health Science, Mattu Univeristy, Mattu, Ethiopia; ^2^Gambella Hospital, Gambella Region Health Bureau, Gambella, Ethiopia; ^3^Pharmacy Department, College of Health Science, Mattu Univeristy, Mattu, Ethiopia; ^4^Ayantu Girma Law Office, Federal and Oromia Region Justice Bureau, Finfinne, Ethiopia; ^5^Department of Population and Family Health Faculty of Public Health, Jimma University, Jimma, Oromia, Ethiopia

**Keywords:** gender-based violence, intimate partner violence, women’s health, women’s rights, Gambella town, Ethiopia

## Abstract

**Abstract:**

**Introduction:**

Intimate partner violence is defined as any behavior by a current or past male intimate partner during marriage, cohabitation, or any other formal or informal union that causes physical, sexual, or psychological harm. Men are the most common perpetrators of this against women. It affects almost one-third of all women worldwide.

**Objective:**

This study aimed to assess the prevalence, consequences, and factors associated with intimate partner violence among partnered women in Gambella town.

**Methods:**

A community-based, cross-sectional study design was employed. A systematic random sampling technique was used to select the study participants. Data was collected using a pretested, structured questionnaire. The data were entered and analyzed using SPSS software version 25. The bivariate and multivariate logistic regression method was used to identify factors associated with intimate partner violence. Variables with a *p*-value <0.05 were considered significantly associated with intimate partner violence.

**Results:**

The overall prevalence of intimate partner violence in the lifetime and the last 12 months was 58.8, 95% CI (54.0, 63.6), and 51.8, 95% CI (46.7, 56.8), respectively. More than half (53.3%) of the violence resulted in physical injury, while 32.9% were separated from their partners whereas, mother’s history of exposure to IPV [AOR: 1.8, 95% CI (1.03–3.27), *p* < 0.05], respondent’s age [AOR: 3.4, 95% CI (1.8, 6.5), *p* < 0.001], substance use [AOR:2.5, 95% CI (1.5–4.1), *p* < 0.001], disagreement on sexual intercourse [AOR:3.2, 95% CI (1.8–5.7), *p* < 0.01], monthly family income [AOR:0.32, 95% CI: (0.16–0.63), *p* < 0.01] and family size [AOR:2.8, 95% CI: (1.6–4.8), *p* < 0.01] were significantly associated with IPV.

**Conclusion:**

The study indicated that the prevalence of intimate partner violence was very high. Age of the woman, family size, substance use, economic status, were among factors significantly associated with intimate partner violence. Therefore, responsible stakeholders should respond to the deep-rooted and highly complicated gender inequality by implementing preventive measures.

## Introduction

Intimate partner violence is defined as any action by a current or past male intimate partner in the context of marriage, cohabitation, or any other formal or informal union that results in physical, sexual, or psychological (1). Intimate partner violence is Crime. It is a violation of women’s human rights (2, 3). Intimate partner violence is mostly perpetrated by men against women (1, 4). It is the most common and prevalent type of violence against women worldwide. (1, 5). It is experienced by over a third of all women worldwide, necessitating immediate action (1). For instance, it is estimated that between 38 and 40% of murders of women are committed by intimate partners (1, 6, 7).

The Least Developed Countries have the highest lifetime Prevalence of intimate partner violence among women aged 15–49. (37%) (8). The highest rate of lifetime intimate partner violence is in Southern Asia (35%) and Sub-Saharan African countries (33%) (1, 9). Furthermore, violence against intimate partners begins early in life. 16% of young women aged 15 to 24 encounter intimate partner violence (IPV) in a year, and nearly 1 in 4 adolescent girls in the 15–19 age cohort are believed to have experienced physical and/or sexual abuse at the hands of an intimate partner (1) (10).

It has substantial immediate, medium, and long-term implications on the well-being of women, children, and families (1) (3). IPV can cause injuries, homicide or suicide, sexually transmitted infections like HIV, stillbirth, pre-term delivery, low birth weight, depression, post-traumatic stress disorder, and other anxiety disorders, sleep problems, eating disorders, increased smoking, substance abuse, risky sexual behavior, and higher infant and child mortality rates. (4, 11, 12)

According to the UNFPA, Africa’s high rate of violence against women and girls (VAW) is due to the continuation of detrimental gender norms, alcohol consumption, and overall increased poverty and conflict (13).

Furthermore, the COVID-19 pandemic and its social and economic consequences have increased women’s exposure to violent partners and other dangers. Measures taken to combat the epidemic, such as lockdowns have led to an increase in reports of domestic violence, in particular intimate partner violence against women. For example, the East African Community (EAC) partner states have reported a sharp (48%) increase in the number of IPV cases during this COVID-19 lockdown have led to an upsurge in reports of domestic violence against women (14). For example, the East African Community (EAC) member states have reported a high (48%) increase in the number of IPV instances during the COVID-19 lockdown (14, 15). The COVID-19 outbreak has also reduced their access to medical care. In addition to the COVID-19 epidemic, situations of humanitarian disasters such as terrible drought and displacements following conflicts or war have aggravated the existing violence and led to new kinds of violence against women (4, 12).

Over the last three decades, many global consensus papers and regional conventions have issued forceful appeals to abolish violence against women. For example, sustainable development goals (SDG) 3 and 5, under their respective targets 7 and 2, respectively, set an agenda for the “elimination of all forms of violence against all women and girls in the public and private spheres, including trafficking and sexual and other types of exploitation, achieving gender equality, and empowering all women and girls.” As such, by the year 2030, every country is expected to be IPV-free (16, 17). Aiming to eliminate IPV, many projects were initiated before and after the establishment of the SDGs (11).

However, it has been recognised that progress has been too slow, and the prevalence of violence against women remains unacceptably high (18).

In Ethiopia, IPV is a major public health concern, a significant challenge, and a threat to women’s empowerment and development (19). In Ethiopia, the lifetime and 12-month prevalence of IPV are estimated to be 37 and 27, respectively (20).

In line with the SDGs, Ethiopia seeks to achieve “zero sexual and intimate partner violence (IPV), including zero child marriage, zero early and forced marriage, and zero female genital mutilation.” To accomplish this, Ethiopia has developed and executed a number legal and policy provisions and initiatives, ratified many international, continental and national agreements, including the Health Sector Transformation Plan II and the National Reproductive Health Strategic Plan, Convention on the Elimination of Discrimination Against Women (CEDAW), the Protocol to the African Charter on the Rights of Women in Africa, the Revised Family Law (in 2000), the Revised Criminal Code (in 2005), (UN Women 2016) and others to protect the rights of women and girls and promote gender equality (19, 21).

However, it has been consistently stated that most African countries (including Ethiopia) do not uphold their pledges as outlined in several international accords and national legal measures. Even though just a few studies have been conducted on intimate partner violence against women in Ethiopia, there is very little (if any) evidence in the study region, particularly in the post-COVID-19 pandemic era, when the frequency of IPV has significantly increased. Furthermore, the research area is marked by negative traditional beliefs and practices like early marriage, child marriage, polygamy, wife inheritance, high HIV prevalence, sociocultural factors such as the use of “Tifo Bet” and social support for polygamy and wife beating, that have many adverse impacts on gender balance (22–24). As a result, this study examined the prevalence and implications of intimate partner violence, as well as the risk variables related with it, among married women in the study area in the aftermath of the COVID-19 epidemic.

## Methods

### Study area and period

This study was conducted from September 17, 2021, to October 15, 2021, in Gambella Town, Southwest Ethiopia. Gambella Town is the capital of the Gambella People’s National Regional State. It is located in the south-western part of the country, 766 kilometres from the capital, Finfinnee/Addis Ababa. The region is bordered to the north, northeast, and east by Oromia National Regional State; to the south and southeast by the Southern Nations and Nationalities People’s Regional State; and to the southwest, west, and northwest by the Republic of South Sudan. As Ethiopia is the third-largest refugee-hosting country in Africa, the Gambella region hosts the largest proportion of refugees, mainly from South Sudan. The town has a total population of 63,357, according to the 2013 Ethiopian Fiscal Year (EFY) population projection. The town has 13,773 households and 16,536 women of reproductive age. In the town, there is one general hospital, one primary hospital, one health centre, five health posts, and 19 private (primary and medium) clinics.

### Study design

A cross-sectional community-based study design was employed.

### Population

#### Source population

A source population consisted of married women aged 15–49 living in Gambella town.

#### Study population

The study population was partnered women aged 15 to 49 who lived in Gambella town and were chosen with systematic random sampling method.

#### Eligibility criteria

##### Inclusion criteria

A woman in the age range of 15–49 years who had been ever a partnered and stayed at least 12 months with her current partner was included in the study.

#### Sample size determination

The sample size was calculated for both objectives (prevalence of intimate partner violence and factors associated with it). For the prevalence of intimate partner violence, the sample size was calculated using a single population proportion formula.


$$\begin{array}{l} n=(Z\alpha /2\left(p\right){\left(1-P\right)}^{2}/d^{2}=\left(1.96\right)2\times \\ \;0.569\left(1-0.569\right)=376.84\sim =377. \end{array}$$

Where, *N* = the sample size; *Z* = 1.96; at 95%CI; *D* = margin of error tolerated (0.05), and *P* = the proportion of IPV against married women from previous study (25).

By adding 5% of non-response, the final sample size became 396. For the second objective (factors associated with IPV), the sample size was calculated using the double population proportion formula by considering frequently associated factors like women’s educational status, women’s supportive attitude toward wife-beating, young age, and alcohol use by male partners from previous studies, and it was 345. But since the sample size calculated for the first objective was larger than the latter, the larger sample size (396) was used in this study.

#### Sampling technique and procedures

**Figure 1:**
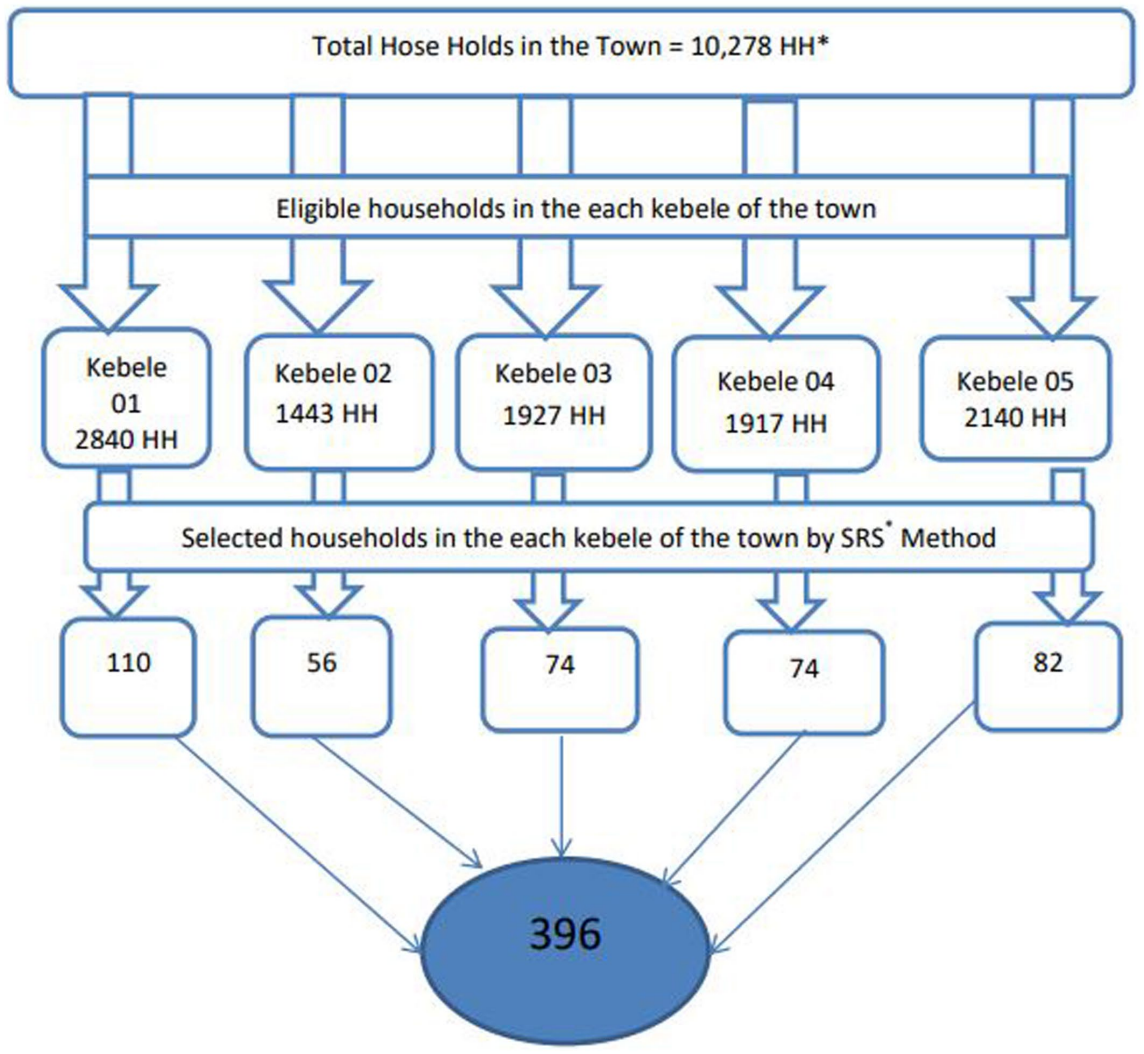
Schematic presentation of sampling procedure to assess IPV among married women in Gambella town, 2021. *HH, house holds; *SRS, simple random sampling.

The town is divided into five kebeles (the basic or smallest administrative unit in the Ethiopian administrative system). All five kebeles in the town were included in the study. A preliminary survey was conducted in each kebeles to identify eligible households and all the eligible houses in each kebel of Gambella town were marked. Then, the number of households to be selected from each kebele was determined using the proportional allocation technique. Participants were drawn from each kebele using systematic sampling methods. The Kth interval was calculated by dividing the source population (N) by the sample size (n) (N/n) and found to be 2. Households were selected using the lottery method to determine which house to start with. Accordingly, every second-eligible house has been interviewed. Multiple eligible women within the same households were also identified by the lottery method. Households that were not available, not interested, or refused to participate were replaced with households that have similar socio-demographic characteristics. See the diagram below (Figure 1).

#### Data collection procedures and data quality control

A pre-tested interviewer-administered structured questionnaire was used to collect data. The questionnaire was tested among 5% of women in itang Woreda which is 40 km away from Gambella town. The results of the pre-testing were used to correct any structural flaws discovered in the tools.

The principal investigator adapted the questionnaire from the WHO multi-country study of VAW (8) and created an English questionnaire, which was then translated into the most commonly spoken local languages (Anyuwa and Nueri and Amharic). theen, it was translated back to English by an other language expert to chech for consistency.

### Data quality control

To ensure data quality, data collectors were chosen from among those with prior experience in the field. Then, they were trained for 2 days. Knowledge of the local language was considered to avoid language barriers. Data collection tools were pre-tested before being sent out into the field. In addition, data collectors and field supervisors checked the completeness and consistency of data in the field on a daily basis. Epi Data Software was used for data entry to reduce potential errors that could occur during data entry.

### Operational definitions

#### Intimate partner violence

In this study, a woman who reported that she had experienced any act of physical, sexual, or emotional (psychological) violence or any combination of the three by an intimate partner was considered to have IPV (1, 26, 27).

#### Physical violence

In this study, respondents were asked if they had experienced one or more acts or threats such as slapping, pushing, shoving, pulling, throwing something that could hurt, choking, burning on purpose, hitting the abdomen with a fist or with something else, and if a gun, knife, or any other weapon was used against the woman by an intimate partner. A woman replied yes to at least one of the following acts and was considered to have experienced physical violence (1).

#### Psychological violence

According to this study, women who replied yes to one or more acts or threats such as insult, humiliation, intimidation, or being scared on purpose by an intimate partner was considered to have experienced psychological violence (1, 26).

#### Sexual violence

Women who replied yes to one or more acts or threats, such as being forced into sexual intercourse when they did not want to, having sexual intercourse when they did not want to because they were afraid of what their partner might do, and being forced to do something sexual that she found degrading or humiliating by an intimate partner was considered to have experienced sexual violence (1, 27).

#### Severe violence

is defined based on the severity of the acts; the following are defined as severe: being beaten up, choked, or burned on purpose, and/or being threatened or having a weapon used against them. Any sexual violence is considered severe (1).

### Data analysis

The data was checked for completeness and consistency. Then it was entered into a computer using Epidata software version 3.1 and exported to SPSS version 22 for cleaning, recoding, and analysis. Descriptive results like percentage and frequency distributions of all variables were presented using tables and charts. Then a univariable logistic regression analysis was run to identify variables that were candidates for the multivariable logistic regression analysis. Accordingly, variables with a *p*-value less than 0.25 in the univariable logistic regression analysis were considered candidates for the multivariable logistic regression analysis. The goodness of the fit of the final model was checked using the Hosmer-Lemeshow test, considering a good fit at a *p*-value >0.05. Then a multivariable logistic regression model was run, and variables with a p-value less than 0.05 at 95% CI were declared to be significant, and adjusted odds ratios were used to measure the strength of the association.

### Ethics, approval, and consent to participate

This study was done according to the Declaration of Helsinki. The Research Ethical Committee of Mattu Univeristy granted an ethical letter of approval with Ref:RPG/021/2013 for this study, and written informed consent was obtained from the participants after the necessary explanation was given on the purpose and benefits of the study, as well as their right to decide whether or not to participate in the study. All the interviews with respondents were conducted with strict privacy. Study participants engaged in the study were informed that they could skip any question they did not want to respond to and could quit the interview if they felt discomfort. Then consent was obtained from the study participants, who were 18 years of age or older. For those who were less than 18 years old, assent was obtained from the participants, but consent was obtained from their representatives based on Article 25 of the Declaration of Helsinki.

## Results

### Socio-demographic characteristics of the respondent

A total of 396 women participated in this study, making the response rate 100%. The respondents’ average age was 29 years (SD ±7 years). In terms of educational attainment, 148 (37.34%) of the participants attended primary school or lower, while 248 (62.6%) attended secondary education and above. Thirty-six (9.1%) of the respondent’s partners attended primary school, while the great majority (90.9%) attended secondary school or higher. The household size was four people on average. Fifty-one (12.9%) of our respondents were pregnant at the time of the survey. Seventy-eight (19.7%) of the study participants and 218 (55.1%) of respondents’ partners reported using at least one substance (Table 1).

**Table 1:** Socio-demographic characteristics of the study participants in Gambella town, 2021 (*n* = 396).

Variables	Categories	Frequency (%)
Age in years	≤19 or less	6 (1.5)
	20–29	236 (59.6)
	30–39	97 (24.5)
	≥40	57 (14.4)
	Total	396 (100%)
Educational status	Illiterates (unable to read and write)	42 (10.6)
	Elementary	106 (26.8)
	High School	144 (36.4)
	High school graduate	104 (26.3)
	Total	396 (100%)
Occupation	Housewife	257 (64.9)
	Daily laborer	6 (1.5)
	Merchant	30 (7.6)
	Private employee	19 (4.8)
	Government employee	84 (21.2)
	Total	396 (100%)
Ethnicity	Agenda	14 (3.5)
	Nuer	147 (37.1)
	Amahara	68 (17.2)
	Oromo	152 (38.4)
	Others	15 (3.8)
	Total	396 (100%)
Education status	Primary or less	148 (37.4)
	Secondary and above	248 (62.6)
	Total	396 (100%)
Religion	Orthodox	150 (37.9)
	Muslim	27 (6.8)
	Protestant	136 (34.1)
	Catholic	84 (17.7)
	Others	14 (3.5)
	Total	396 (100%)
Substance use	Yes	78 (19.7)
	No	318 (80.3)
	Total	396 (100%)
Family size (mean = 4)	Less than 4	179 (45.2)
	4 and above	217 (54.8)
	Total	396 (100%)
Mean monthly family income (mean = 5,000 ETB)	5,000 or less ETB	335 (84.6)
	Above 5,000 ETB	61 (15.4)
	Total	396 (100%)
The husband has another wife.	Yes	112 (28.3)
	No	284 (71.7)
	Total	396 (100%)
Pregnancy (Currently)	Yes	51 (12.9)
	No	345 (87.1)
	Total	396 (100%)
Husband education	Primary or less	36 (9.1)
	Secondary and above	360 (90.9)
	Total	396 (100%)
Husband age category	29 years or less	58 (14.6)
	Above 29 years	338 (85.4)
	Total	396 (100%)
Husband occupation	Farmer	21 (5.3)
	Daily laborer	56 (14.1)
	Merchant	69 (17.4)
	Privet institution/NGO	82 (20.7)
	Government employed	168 (42.4)
	Total	396 (100%)
Husband Substance Use	Yes	218 (55.1)
	No	178 (44.9)
	Total	396 (100%)
The husband has another wife (polygamy).	Yes	112 (28.3)
	No	284 (71.7)
	Total	396 (100%)
Attitudinal acceptance of wife-beating among women	Yes (accept)	129 (32.6)
	No	267 (67.4)
	Total	396 (100%)

In this study, 122 (28.3%) respondents reported that they had a spouse with another wife (a polygamist husband), and 129 (32.6%) believe that wife battering is appropriate (believe that their husband has the right to beat them) (Table 1).

### Prevalence of intimate partner violence (IPV)

#### Lifetime and the last 12 months prevalence of intimate partner violence

The overall prevalence of intimate partner violence (IPV) in the lifetime and in the last 12 months was 58.8, 95% CI (54.0, 63.6), and 51.8, 95% CI (46.7, 56.8), respectively.

### The commonest form of intimate partner violence

The commonest form of IPV was Psychological/emotional violence (168, 42.4%), followed by sexual violence (154, 38.9%) and physical violence (74, 18.7%), respectively (Table 2). Among the respondents, 256 (64.6%) have a history of IPV during their childhood, 233 (58.8%) have had lifetime IPV from someone after marriage, and 212 (53.5%) reported that their mother encountered IPV from their father. Moreover, nearly half (44.9%) of the study participants had encountered severe forms of violence (Table 2).

**Table 2:** Prevalence, forms, and consequences of IPV among study participants in Gambella town, southwest Ethiopia, 2021.

Variable characteristics of IPV		Frequency	Percentage %
Sexual violence in the last 12 months		154	38.9
Physical violence in the last 12 months		74	18.7
Psychological and emotional violence in the last 12 months		168	42.4
Total IPV in the last 12 months in any form		205	51.8
Lifetime exposure to IPV		233	58.8
History of IPV during childhood or before marriage from family or others		256	64.6
Mother’s history of IPV: whose mother encountered IPV		212	53.5
Sever Violence		178	44.9
Consequences of IPV on families couples	Physical injury with IPV	113	53.3
	Separated because of a IPV	26	32.9

### Consequences of IPV among study participants

Of the total number of women who participated in this study, 139 (59.7%) suffered from one or more consequences. One hundred thirty-nine (48.5%) of the women who had IPV had suffered from different types of physical injuries. Similarly, twenty-six (11.2%) of IPV incidences resulted in divorce (Table 2).

### Factors associated with intimate partner violence (IPV)

In univariable analysis, the following variableswere considered candidates for multivariable logistic regression analysis: pregnancy, maternal history of exposure to IPV, support from other victims of IPV, age of the respondent, substance use by respondents, substance use disagreement on sexual intercourse, monthly family income, and family size were identified and taken to multivariable logistic regression analysis.

In multivariable logistic regression analysis (Table 3), the history of exposure to IPV during childhood, substance use by respondents, husband or partner substance use, disagreement on sexual intercourse, monthly family income, and family size were found to be significantly associated with intimate partner violence among the study participants.

**Table 3:** Factors associated with intimate partner violence among married women aged 15–49 in Gambella town during 2021 in southwest Ethiopia.

Factors		IPV		COR	AOR	95% CI	*P*-value
Variables	Categories	Yes	No				
Pregnancy	Yes	35	16	2.25	0.97	0.46–2.06	0.945
	No	170	175	1	1		
Maternal history of exposure to IPV	Yes	149	107	2.09	1.83	1.03–3.27	0.04*
	No	56	84	1	1		
Support for any one victim of IPV	Yes	80	49	1.85	1.8	0.99–3.26	0.053
	No	125	142	1			
disagreement on sexual intercourse	Yes	81	32	3.25	3.22	1.82–5.7	<0.001**
	No	124	159	1	1		
Mean monthly family income (ETB)	<= 5,000 ETB	160	175	0.32	0.315	0.158–0.62	0.001**
	>5,000 ETB	45	16	1	1		
Family size	> = 4 member	108	71	1.89	2.798	1.61–4.85	<0.001**
	<4 member	97	120	1	1		
Husband’s substance use	Yes	144	74	3.73	2.458	1.48–4.06	<0.001**
	No	61	117	1	1		
Substance use by the respondent	Yes	59	19	3.66	3.42	1.79–6.50	<0.001**
	No	146	172	1	1		
Age of women in years	19 and below	3	3		1		
	20–29	115	121	1.052	0.195	0.032–1.186	0.076
	30–39	61	36	0.590	0.101	0.015–0.672	0.078
	40 and above	26	31	1.192	0.273	0.040–1.869	0.186

The odds of intimate partner violence among women who had a childhood history of exposure to intimate partner violence increased by 80% [AOR: 1.8, 95% CI (1.03–3.27), *p* < 0.05] when compared to women who had no childhood history of exposure to violence.

Women who had disagreements with their husbands on sexual intercourse were 3.2 times more likely to be victims of IPV [AOR: 3.2, 95%CI (1.8–5.7), *p* < 0.01] as compared to women who had no disagreements with their husbands on sexual intercourse.

Women who had an estimated monthly income of more than five thousand Ethiopian Birr were 68% less likely to face Intimate partner violence [AOR: 0.32, 95%CI (0.16–0.63), *p* < 0.01] than those who had an estimated monthly income higher than that.

Women with a family size of four or above were 2.8 times more likely to be victims of intimate partner violence [AOR: 0.32, 95%CI (0.16–0.63), *p* < 0.01] as compared with those who had less family size.

Those women whose husbands use at least one substance were 2.5 times more likely to be the victims of intimate partner violence [AOR: 2.5, 95% CI (1.5–4.1), *p* < 0.001] than those women whose husbands do not use a substance.

Women who use at least one substance were 3.4 times more likely to be victims of substance use [AOR: 3.4, 95% CI: (1.8–6.5); *p* < 0.001] than those who do not use a substance (Table 3).

## Discussion

This study identified both the overall prevalence, forms of intimate partner violence, and consequences of intimate partner violence against women, as well as associated factors, in one of the peripheral parts of Ethiopia, the Gambella region. The overall prevalence of intimate partner violence against women was very high.

This finding is in line with the findings of different studies conducted in Ethiopia (47%) (28) (51.7%) (29), (48.6%) (30), in Bangladeshi (53%) (31), and in Nigeria (50%) (32). On the other hand, this finding is higher than studies conducted in Ethiopia (32.5%) (11), (19.6%) (33) in sub-Saharan Africa (35.5%) (34), in Nigeria (29%) (35), in the Republic of Cyprus (8, 36).

This may be due to the differences in socio-cultural context and the scope of these studies; the study conducted in sub-Saharan Africa is a pooled prevalence that covered a large geographical area and a huge sample size. Similarly, the study conducted in the Republic of Cyprus was a national survey that included a large sample size. Furthermore, the difference might be attributed to the better socio-economic and socio-cultural conditions (better handling of human rights) in Cyprus as compared to Ethiopia.

The finding of this study is lower than the studies conducted in southwest Ethiopia (64.7%) (26), northwest Ethiopia (78%) (37), and Lublin, Poland (59.7%) (38).

This could be because these studies were conducted in rural communities in peripheral Ethiopia, where there is poor awareness about gender equality and wife beating is more widely accepted as a normal phenomenon when compared to urban residents.

This study indicated that all forms of violence against women, including the severer ones, are highly prevalent. This study also showed that the gap between the lifetime and current prevalence of violence against women in the study area is very short—58.8% versus 51.8%, respectively. This means gender-based violence is deeply responding to interventions or there were no sounding interventions, in the region. In other words, women in these areas will continue to live deprived of their human rights.

The following variables were found to be significantly associated with intimate partner violence: the history of exposure to IPV during childhood, substance use by respondents, substance use by husband or partner, disagreement on sexual intercourse, monthly family income, and family size.

The odds of intimate partner violence among women who had a childhood history of exposure to violence increased by 80% when compared to women who had no childhood history of exposure to intimate partner violence. This is similar to studies conducted in Ethiopia (29, 39), Uganda (40), and India (41).

This could be because women who has history of exposure to violence might have accepted being beaten as a norm phenomenon (attudinal acceptance of wife-beating) Women who had an estimated monthly income of more than five thousand Ethiopian birr were 68% less likely to face intimate partner violence than those who had an estimated monthly income lower than that. This finding is in line with studies conducted in Ethiopia (systematic review) (29) but against a study conducted in Ethiopia (25) and China (42).

This could be because those women who have a higher income might also have a higher education status, better economic autonomy, and use all the available opportunities to defend themselves against violence.

Women with a family size of four or above were 2.8 times more likely to be victims of intimate partner violence as compared with those who had a smaller family size. This finding is consistent with studies done in northwest Ethiopia (30) and Turkiye (43) but against a study conducted in Bangladesh (44) which reported that there is no association between IPV and family size. This could be attributed to the reality that families with larger family sizes would have difficulty providing necessities for family members and may result in disagreements in prioritising family expenditures, which in turn increases the likelihood of conflict between partners.

Those women whose partner use at least one substance were 2.5 times more likely to be victims of intimate partner violence than those whose husbands do not use a substance.

This finding is consistent with a systematic review study conducted in African countries (45), and multiple studies done in Ethiopia (20, 27, 29, 46, 47). This can be attributed to the effect of alcohol consumption on cognitive capability: it alters the self-control ability of individuals, results in poor judgment, and triggers the individual to act aggressively.

Women who use at least one substance were 3.4 times more likely to be victims of intimate partner violence than those who do not use substances. This finding is in line with studies conducted in Ethiopia (29, 32). This is similar with the effects of alcohol consumption mentioned above,.

Women who had disagreements with their husbands on sexual intercourse were 3.2 times more likely to be victims of IPV as compared to their counterparts. This could be because male partners might use the advantage of power inequality between males and females and use force to have sexual intercourse.

Consequently, several previous research frequently referenced the same traits that this study found, including substance abuse, family income, and a history of being exposed to intimate partner violence as a youngster. The authors’ analysis, however, could not find any indication of the factor “disagreement on sexual intercourse” in previous study.

Furthermore, the findings have major policy consequences. Intimate partner violence is not only a serious public health issue, but it also violates fundamental human rights. To put it plainly, IPV is a felony. For example, intimate partner violence (gender-based violence in general) violates multiple women’s human rights provided in the Ethiopian constitution, such as “The Right of the Security of Person” (Article 16), “Prohibition against Inhuman Treatment” (Article 18), “Right to Honour and Reputation” (Article 24), “Right to Equality” (Article 25), and “Rights of Women” (Article 35) (48, 49).

At the same time, it is a major development concern. However, it appears that women’s issues, specifically IPV, are not receiving the attention they need.

The Sustainable Development Goal (SDG) 5 (under Targets 1 and 2) states, “End all forms of discrimination against all women and girls everywhere; eliminate all forms of violence against all women and girls in the public and private spheres, including trafficking and sexual and other forms of exploitation” (17).

However, it appears that the opposite is occurring. In Africa, for example, intimate partner violence has grown by 48% since the start of the COVID-19 pandemic [11]. Furthermore, drought and conflict disproportionately impact women. In Ethiopia, the terrible drought, extensive conflicts, and the COVID-19 pandemic have all affected women’s sexual and reproductive health, exacerbating IPV. Rape has long been used as a weapon of war in conflict zones. To summarize, this study attempted to assess the prevalence of intimate partner violence in war-torn Ethiopia amid the COVID-19 epidemic, a nationwide political crisis, conflicts, and a severe drought.

This study’s strengths include conducting a community-based investigation and gathering primary data.

This study, however, has certain limitations. Intimate relationship violence is a difficult subject. It is a legal matter. It is about the loved one of many women who are in marriage. As a result, many of them may be reluctant to reveal the truth, either out of fear or as a commitment to maintaining family secrets.

The fact that this study only included partnered women as participants is another drawback. Therefore, IPV among people who are not in formal relationships was not addressed in this study. As a result, the prevalence of IPV may be higher than what this study found. Furthermore, this study did not assess the fate of the victims of the IPV. Specially women who sustained severe violence, like sexual violence, were expected to receive medical, psychological, and legal remedies. However, these points were beyond the scope of this study.

Since this study is a cross sectional study, it is not possible to establish causal inference in this study. Finally, this study could have been more informative if it had been accompanied by a qualitative study.

## Conclusion

All the forms of violence—sexual, psychological, and physical violence (including severe violence)—against women were very common, and they had far-reaching consequences. This history of exposure to IPV during childhood, substance use by respondents, substance use by partners, ignorance due to sexual intercourse disagreement, monthly family income, and family size were identified as determinants of intimate partner violence (IPV). The responsible government and non-government organisations should respond to the deep-rooted and highly complicated gender inequality by implementing preventive measures and providing comprehensive rehabilitative services for the survivors of IPV. Additionally, interested future researchers are highly encouraged to conduct a study that addresses the limitations listed above by considering longitudinal studies to establish causal relationships and qualitative studies to provide a more in-depth understanding of the context and drivers of IPV.

## Recommendations

### For the Gambella regional health office, the women, children, and youth affairs office, and other stakeholders (NGOs)

- Should work to create awareness and/or strengthen preventive action on women’s (gender) equality to promote mutually respectful relationships among the couples;
- Safeguard women’s human rights and respond to deep-rooted gender inequality by making the perpetrators accountable whenever and wherever it happens.
- Since the prevalence of intimate partner violence is very high with complicated outcomes, we recommend that the regional health office establish a one-stop centre in the area and provide comprehensive rehabilitation services to the survivors of intimate partner violence.

### For the local healthcare workers

- Health workers should counsel women on gender equality and the way out when it happens to all women who visit health facilities for any reason and through community outreach, whoever the perpetrator is, regardless of the condition it happened in.

### For the future researchers

- We recommend future researchers interested in conducting research in this field address the limitations stated above:
- Included all women in the reproductive age group (15–15), regardless of their marital status.
- Supporting a qualitative study or running a pure qualitative study

## Data availability statement

The original contributions presented in the study are included in the article/supplementary material, further inquiries can be directed to the corresponding author.

## Ethics statement

The studies involving humans were approved by the Research Ethical Committee of Mattu Univeristy. The studies were conducted in accordance with the local legislation and institutional requirements. The participants provided their written informed consent to participate in this study.

## Author contributions

AbG: Conceptualization, Data curation, Formal analysis, Funding acquisition, Investigation, Methodology, Software, Supervision, Validation, Visualization, Writing – original draft, Writing – review & editing. TA: Conceptualization, Data curation, Formal analysis, Funding acquisition, Investigation, Methodology, Project administration, Resources, Software, Visualization, Writing – original draft, Writing – review & editing. DC: Conceptualization, Data curation, Formal analysis, Investigation, Methodology, Supervision, Validation, Writing – original draft, Writing – review & editing. GD: Funding acquisition, Investigation, Methodology, Writing – original draft, Writing – review & editing. AyG: Formal analysis, Investigation, Validation, Visualization, Writing – original draft, Writing – review & editing. MA: Conceptualization, Data curation, Formal analysis, Investigation, Methodology, Software, Supervision, Validation, Writing – original draft, Writing – review & editing.
